# Antiviral Activity and Molecular Dynamics Simulation of Hops Compounds against Oropouche Virus (*Peribunyaviridae*)

**DOI:** 10.3390/pharmaceutics15122769

**Published:** 2023-12-13

**Authors:** Tsvetelina Mandova, Marielena Vogel Saivish, Gabriela de Lima Menezes, Katyanna Sales Bezerra, Umberto Laino Fulco, Roosevelt Alves da Silva, Fernando Batista Da Costa, Maurício Lacerda Nogueira

**Affiliations:** 1 AsterBioChem Research Team, School of Pharmaceutical Sciences of Ribeirão Preto, University of São Paulo, Ribeirão Preto 14040-020, SP, Brazil; 2 Gilson Purification, 22 rue Bourseul, 56890 Saint Avé, France; 3 Laboratórios de Pesquisas em Virologia, Departamento de Doenças Dermatológicas, Infecciosas e Parasitárias, Faculdade de Medicina de São José do Rio Preto, São José do Rio Preto 15090-000, SP, Brazil; marielenasaivish@gmail.com (M.V.S.);; 4 Brazilian Biosciences National Laboratory, Centro Nacional de Pesquisa em Energia e Materiais (CNPEM), Campinas 13083-100, SP, Brazil; 5 Bioinformatics Multidisciplinary Environment, Programa de Pós Graduação em Bioinformática, Universidade Federal do Rio Grande do Norte, Natal 59078-400, RN, Brazil; gabrieladelima1804@gmail.com (G.d.L.M.); umberto.laino@ufrn.br (U.L.F.); 6 Núcleo Colaborativo de Biosistemas, Universidade Federal de Jataí, Jataí 75801-615, GO, Brazil; roosevelt@ufj.edu.br; 7 Department of Pathology, University of Texas Medical Branch, Galveston, TX 77555, USA

**Keywords:** bunyavirales, cap-snatching, oropouche virus, endonuclease, natural products, hops, acylphloroglucinols, xanthohumol, in silico method, molecular dynamics simulation

## Abstract

The Oropouche virus (OROV) is a member of the family *Peribunyaviridae* (order *Bunyavirales*) and the cause of a dengue-like febrile illness transmitted mainly by biting midges and mosquitoes. In this study, we aimed to explore acylphloroglucinols and xanthohumol from hops (*Humulus lupulus* L.) as a promising alternative for antiviral therapies. The evaluation of the inhibitory potential of hops compounds on the viral cycle of OROV was performed through two complementary approaches. The first approach applies cell-based assay post-inoculation experiments to explore the inhibitory potential on the latest steps of the viral cycle, such as genome translation, replication, virion assembly, and virion release from the cells. The second part covers in silico methods evaluating the ability of those compounds to inhibit the activity of the endonuclease domain, which is essential for transcription, binding, and cleaving RNA. In conclusion, the beta acids showed strongest inhibitory potential in post-treatment assay (EC_50_ = 26.7 µg/mL). Xanthohumol had the highest affinity for OROV endonuclease followed by colupulone and cohumulone. This result contrasts with that observed for docking and MM/PBSA analysis, where cohumulone was found to have a higher affinity. Finally, among the three tested ligands, Lys92 and Arg33 exhibited the highest affinity with the protein.

## 1. Introduction

**Figure 1:**
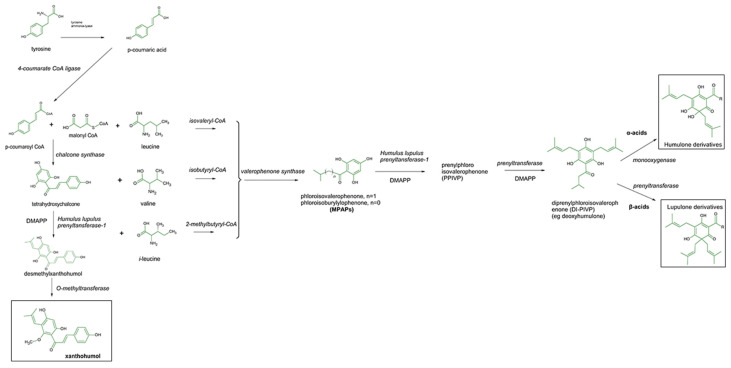
Proposed mechanism for the enzymatic formation of the three group of studied compounds (framed): phloroglucinols (humulone and lupulone) and xanthohumol, after two pathway of biosynthesis, adapted after [13] (DMAPP—dimethylallyl pyrophosphate); italics indicate the enzymes involved in the pathway.

The order *Bunyavirales* contains potentially fatal viruses that lack effective medical countermeasures. Many of them pose major public health threats and are pathogens prioritized in the World Health Organization (WHO). Their hallmark is their segmented single-stranded RNA (ssRNA) genome with either negative or ambisense polarity. Thus, the synthesis of viral mRNA can be considered a limiting step in the early stages of the life cycle. The presence of 5′-capped extensions derived from the host mRNA in the viral transcripts suggests the use of a cap-snatching mechanism to initiate transcription. This mechanism was initially described for the Influenza virus (IFV), and, although the specifics might vary between viruses, a constant feature is a metal dependent cap-snatching endonuclease responsible for the mRNA cleavage [1,2]. Bunyaviruses are also known to have a cap-dependent endonuclease domain at the N-terminus of the L protein (2100–2900 residues, except *Bunyaviridae*) [3]. These cytoplasmically replicating viruses include several emerging, vector-borne human pathogens, e.g., Lassa virus (LASV), Crimean-Congo hemorrhagic fever virus (CCHF), La Crosse virus (LACV), Severe Fever with Thrombocytopenia Syndrome virus (SFTSV), and Oropouche virus (OROV), amongst others. The latter is the causative agent of Oropouche fever [4]. The transmission occurs by the bite of infected *Culicoides paraensis* midges; however, its first isolation occurred in 1955 from a human case in the village of Vega de Oropouche in Trinidad related to *Coquillettidia venezuelensis* mosquitoes [5]. OROV belongs to the *Orthobunyavirus oropoucheense* species, *Peribunyaviridae* family, *Orthobunyavirus* genus, and Simbu serogroup. Its prevalence in the Americas, especially in Central and South America, has made it a potential candidate for epidemics and outbreaks [6]. Despite the knowledge on its epidemiology and geographical distribution and circulation, OROV is still a neglected tropical disease with great potential to cause future epidemics and spillover events in Neotropical areas, especially due to challenges in clinical diagnosis, which depends mostly on commercially unavailable serological assays, and therefore, to this date, no licensed vaccination is available yet [6]. All this still makes the search for antiviral molecules and their effects on viral infection as well as viral elements an important field to develop future antiviral therapies. The endonuclease, an enzyme involved in the cap-snatching mechanism, has two divalent metal ions at its active center. The enzyme exhibits nuclease activity and depends on compounds that chelate metal ions at the active center, and therefore it can inhibit enzymatic reactions and exert antiviral activity [3,7]. At the same time, several drugs for treating a variety of infectious diseases have been discovered after the screening of compounds from natural sources, obtained from marine organisms, microorganisms, or plants. In this context, monocyclic polyprenylated acylphloroglucinols (MPAPs) are a significant group of characteristic natural products, featuring highly oxygenated acylphloroglucinol cores densely substituted by prenyl or geranyl side chains. Acylphloroglucinols are a broad class of compounds structurally derived from 1,3,5 trihydroxybenzenes (phloroglucinol) and characterized by the presence of a CRO group (acyl group) and are mainly found in hops. The female inflorescences (hop cones or “hops”), rich in polyphenolic compounds and acyl phloroglucides, are widely used to preserve beer and they provide a characteristic mechanism of microbial inhibition [8]. Figure 1 gives an overview of enzymatic formation in the hop cones of the three groups of studied compounds. Many of the acylphloroglucinols are found in natural sources and exhibit a variety of biological activities: antioxidant, antimalarial, anticancer, antituberculosis, antifungal, and others [8,9]. Their medicinal properties make them interesting candidates as lead structures for drug development. Xanthohumol exhibits significant effects in combating aging, diabetes, inflammation, and microbial infections [10]. This prenylated chalcone has demonstrated potential benefits against breast cancer in in vivo and bioassay experiments. This mechanism is achieved by reducing the levels of anti-apoptotic proteins such as Bcl-2 and procaspase 3 while increasing the presence of pro-apoptotic protein Bax. Additionally, xanthohumol enhances markers associated with DNA damage such as γ-H2AX and cleaved PARP [11]. Considering these characteristics, we were motivated to explore the antiviral activity of the major hop compounds. Our previous research into the antiviral properties of key compounds—acylphloroglucinols and xanthohumol, derived from *Humulus lupulus* L. (Cannabaceae) against the chikungunya virus, showed highly promising results [12]. This widely recognized ingredient in the global brewing industry inspired us to further explore the potential antiviral effects of these major compounds against various types of viral strains. Additionally, we aimed to conduct a more comprehensive investigation into the potential mechanisms of inhibition, utilizing in silico analysis. These results instigated us to evaluate in vitro its viral progeny yields by plaque formation assays on the courses of 12, 24, and 48 h post-infection periods on infected mammal cells. Computational modeling studies (docking and molecular dynamics) were also conducted to explore the binding attitudes of some model hops ligands within the active site of cap-dependent endonuclease.

## 2. Materials and Methods

### 2.1. Cell Culture and Virus Stock

Vero cells were culture grown in Minimal Essential Medium (MEM) (Gibco, Waltham, MA, USA) supplemented with 10% (*v*/*v*) heat-inactivated fetal bovine serum (FBS) (Gibco, Waltham, MA, USA), 100 U. mL^−1^ of penicillin, 0.1 mg. mL^−1^ of streptomycin, and 0.5 µg. mL^−1^ of amphotericin B (Gibco, Waltham, MA, USA) and incubated at 37 °C under a humidified atmosphere containing 5% CO_2_. The C6/36 cells were culture in Leibovitz-15 medium (L-15) with a 10% FBS at 28 °C. Brazilian prototype strain of Oropouche virus (strain Belém prototype BeAn 19991) [14] stock propagated in C6/36 cells and titrated in Vero cells using a plaque-forming assay (PFU), described below.

### 2.2. Cytotoxicity Analysis

The present methodology was executed following established procedures, as previously reported [12,15,16]. In brief, Vero cells (4 × 10^4^/well) were cultured in 96-well plates with MEM and subjected to treatment with each of the five fractions obtained after CCC (countercurrent chromatography) purification, a liquid–liquid chromatography technique. Concentrations ranging from 31.25 µg/mL to 250 µg/mL were applied for 72 h to evaluate the impact of these fractions on cell viability. After this incubation period, 3-(4,5-dimethyl-2-thiazolyl)-2,5-diphenyltetrazolium bromide (MTT, Sigma-Aldrich, Saint Louis, MI, USA) at a concentration of 1 mg/mL was added to the cells, and they were incubated for an additional hour. Subsequently, DMSO was used to dissolve the formazan crystals, and absorbance was measured at 550 nm using a Spectramax Plus Microplate reader (Molecular Devices, Sunnyvale, CA, USA). The results are presented as the percentage of viable cells relative to untreated control cells. Each assay was independently performed three times with triplicate samples. The effective concentration of 50% inhibition (EC_50_) was determined through a dose–response curve using GraphPad Prism (version 8.00) with a four-parameter curve-fitting analysis.

### 2.3. Viral Infection Assay

After treatment with compounds or DMSO, viral replication and titers were determined by plaque assays of culture supernatants, respectively, at 12 to 48 h post-infection. The objective of this treatment during the viral post-inoculation period was to assess the antiviral effects of hops fractions during the viral post-entry stages. Experiments were conducted using Vero cells that were seeded in 24-well plates (2 × 10^5^/well). Initially, the cells were infected with an initial multiplicity of infection (MOI) of 0.1 with OROV and incubated for 1 h at 37 °C, after which the inoculum was removed. Subsequently, the cells were treated with hops fractions, DMSO (used as a vehicle control), or MEM supplemented with 2% (*v*/*v*) FBS (used as a viral control). At specified time points (12, 24, or 48 h post-infection), cell supernatants were collected to determine viral titers using a plaque-forming assay, as described below. The range tested was 125 µg/mL to 31.25 µg/mL. This procedure was repeated in three independent experiments. Data were subjected to analysis using a four-parameter curve-fitting on a dose–response curve through GraphPad Prism (version 8.00) to calculate the EC_50_. The selectivity index (SI) for each of the compounds was determined as the ratio of CC_50_ to EC_50_.

### 2.4. Virus Plaque-Forming Units Assay

Briefly, Vero cells grown in a 24-well culture plate (2 × 10^5^/well) were infected by 0.1 mL of ten-fold dilutions of supernatants. Following an incubation of 1 h at 37 °C, 0.5 mL of culture medium supplemented with 2% fetal bovine serum (FBS) and 1.5% carboxymethylcellulose sodium salt (Sigma-Aldrich, Saint-Quentin-Fallavier, France) were added, and the incubation was extended for three days at 37 °C. The cells were fixed (formaldehyde 10%) and stained with 2% crystal violet diluted in 20% ethanol after removing the media. Plaques were counted and expressed as plaque-forming units per milliliter (PFU·mL^−1^).

### 2.5. Ensemble Docking, Molecular Dynamics (MD) Simulations, and Binding-Free Energy Analysis

#### 2.5.1. 3D Modelling of OROV N-Terminal OROV Endonuclease

The N-terminal OROV Endonuclease domain was represented as a 3D model in this study. However, due to the unavailability of the crystallographic structure, the N-terminal sequence (1–180) corresponding to the endonuclease domain of the polymerase protein was retrieved from the GenBank database (accession code: AJE24678) for the purpose of molecular modeling, which was accomplished using the I-TASSER 5.1. server (https://zhanggroup.org/I-TASSER/, accessed on 20 February 2023) To evaluate the model’s quality, the initial model’s coordinates were assessed using the MolProbity server [17]. In order to include the manganese ion (Mn^2+^) in the model, it was integrated by superimposing the N-terminal endonuclease structure of La Crosse virus (PDB ID: 2XI5), as it was missing in the output model. Unfortunately, the experimental model only contained a single ion, whereas the literature described the presence of two ions. The structure PBD ID: 2XI7 [18], despite having two Mn^2+^ ions, had a ligand attached to the site, which could interfere with the coordination of the Mn^2+^ ions. For this reason, it was decided to adopt the coordinates of the ion from the structure 2XI5, which lacked a ligand. Subsequently, the PropKa 2.0. server (https://www.ddl.unimi.it/vegaol/propka.htm, accessed on 22 February 2023) [19] was employed to predict the protonation state of the amino acid side chains at pH 7.4.

##### 2.5.2. Molecular Dynamics Simulation of N-Terminal OROV Endonuclease

Molecular dynamic (MD) simulations without ligands were conducted to investigate the behavior of the N-terminal OROV Endonuclease domain prior to ligand docking. A system was prepared and subjected to MD simulations for 200 ns each using GROMACS 2020.3 software [20] with Amber ff99SB-ILDN as the force field. The system was placed in a cubic box with the TIP3P water model extended 12 Å away from solute atoms and neutralized with Na^+^ ions. To eliminate bad contacts in the initial structure, two rounds of energy minimization were performed. The first step of minimization involved 500 steps as maximum or until the maximum force on any atom dropped below 50 kJ/mol/nm. For the latest, protein restraint with the steepest descent algorithm, focusing on solvent relaxation was used. The second step of minimization was carried out in flexible water, without protein restraint, using the same steepest descent algorithm. This time, the increase to 10,000 steps was made as the maximum value, or they were applied until the force on any atom fell below 250 kJ/mol/nm.

The pressure of the system was defined to 1 atm, and the temperature was adjusted to 310 K, in two separate 100 ps steps. As reference were used the temperature setting (NVT ensemble) and the pressure setting (NPT ensemble). The modified Berendsen [21] and Parrinello–Rahman [22] algorithms were applied to control the system temperature and pressure, respectively. Hydrogen bonds were constrained using the LINCS algorithm, for the both steps, [23,24] and positional restraints were applied to the protein to stabilize the solvent around the solute.

The Particle Mesh Ewald summation method was applied to calculate the long-range electrostatic interactions, with a non-bonded interaction cut-off of 1 nm. The equations of motion were integrated using the leap-frog algorithm [24] with a time step of 0.2 fs. Before the MD simulation, a 1 ns small NPT ensemble was performed without any restrictions on protein position, and the production run was conducted for 200 ns without any limitation on protein conformation. From the MD run, 2000 protein frames were generated, in total.

##### 2.5.3. Ensemble Docking

AutoDock Vina 1.2.2 software (referred to here only as Vina) was used for docking. First, the 3D structure of the ligands (x = alpha- or beta-acids or xanthohumol), were retrieved from the PubChem database (https://pubchem.ncbi.nlm.nih.gov/compound/639665, https://pubchem.ncbi.nlm.nih.gov/compound/373677 and https://pubchem.ncbi.nlm.nih.gov/compound/196915, accessed on 5 March 2023) in SDF format. The molecule was converted to PDB format using Marvin Sketch software 14.9.22.0. OpenBabel software 3.1.0. was used to convert PDB to PDBQT, setting the pH of the ligand to 7.4.

Ensemble docking was performed using all frames (2000 total) from the MD run obtained in the previous step. To perform the conversion of all frames from PDB to PDBQT, the prepare_receptor.py script from the MGLTools 1.5.7. package was used. The grid box to delimitate the ligand pose searching was based on all frames of trajectory. The binding pocket is the same as the binding of Mn^2+^, so the grid box was defined as the smallest that encompassed this pocket in all protein frames. The Vina output contained 10 binding poses for each frame–ligand docking. The best score (in kcal/mol) for each frame was recorded and analyzed in RStudio software, 4.1.1 software.

##### 2.5.4. Molecular Dynamics (MD) Simulations and MM/PBSA Free Energy Calculation

The complexes of colupulone, cohumulone, and xanthohumol were evaluated using Vina docking, and the best scores were selected for subsequent MD complex analysis. MD simulations were performed in independent triplicate using GROMACS 2022.2 software [25]. Ligand parameters for MD simulations were determined using the ACPYPE 2022.7 server (https://www.bio2byte.be/acpype/) assessed on 13 March 2023 [26], with BCC as the charge method and GAFF2 as the atom type. The server provided all topology and parameter files required for MD simulation applying GROMACS 2022.2 software. Nine MD simulations (three for each compound) of the protein–ligand complex were performed. The MD parameters were the same as MD for protein-only. A total of 2000 frames were made for each MD run.

Using the gmx_MMPBSA 1.6.1. program [27] for each MD, the last 500 frames (50 ns) of the complexes were analyzed using Poisson–Boltzmann surface area (MM/PBSA) free energy calculation, with all solvent molecules and Mn^2+^ ions removed, and the internal dielectric constant was set to 4 (indi = 4), which is the most accurate for highly charged binding pockets, according to comparative studies [28].

The MD trajectories were visualized using UCSF Chimera 1.14. software [29]. The root mean square deviation (RMSD) and fluctuation (RMSF) were calculated using the “gmx” commands of the GROMACS 2022.2 package. All plots were generated using the R language in Rstudio 4.1.1 (http://www.rstudio.com/, assessed on 13 March 2023), and protein image representations were created using UCSF ChimeraX 1.3. [30].

##### 2.5.5. Quantum-Mechanical Analysis of Binding Energy 

The complexes that exhibited lower binding energy, according to the MM/PBSA analysis, were further subjected to quantum-mechanical binding energy calculations using the Density Functional Theory (DFT) formalism, in combination with the molecular fragmentation with conjugate caps (MFCC) approach.

To check the protonation states of the proteins and ligands in 7.4 pH, the PROPKA server and MarvinSketch code version 17.24 (Marvin Beans Suite—ChemAxon, https://www.chemaxon.com, assessed on 13 March 2023) were used. The geometry of the atoms was optimized by subjecting the system to a classical energy minimization (EM) step using the CHARMm (Chemistry at Harvard Molecular Mechanics) force field [31,32], with the protein backbone being constrained. The EM process employed the Smart Minimizer algorithm and was carried out with convergence tolerances set to 10^−5^ kcal mol^−1^ (total energy change), 10^−3^ kcal mol^−1^ (mean square root of the RMS gradient), and 10^−5^ Å (maximum atomic displacement).

To decrease the computational cost of QM calculations, we utilized the molecular fragmentation with conjugate caps (MFCC) approach [33], which involved splitting the protein into individual amino acids by breaking the peptide bonds. To complete the opened valence in the N- and C-terminals, we included the amino acid residues that preceded and succeeded the main residue in the calculation, referred to as “caps”. This approach enabled the system to be calculated in parts instead of as a whole, considerably reducing computational time. Equation (1) shows the MFCC scheme for calculation of interacting energy between ligand and amino acid (IE(LIG/R^i^)).

IE(LIG/Ri) = E(LIG + C^i−1^R^i^C^i+1^) − E(C^i−1^R^i^C^i+1^) − E(LIG + C^i−1^C^i+1^) + E(C^i−1^ C^i+1^) (1)

In order to calculate the interaction energy between the ligand and the main residue, the protein was fragmented into individual amino acids by breaking the peptide bonds. The ligand is labeled as LIG and the main residue as R^i^, where i denotes the ith amino acid of protein chain. To account for the neighboring residues covalently bound to the amine and carboxyl group of R^i^, two caps were introduced, C^i−1^ and C^i+1^.

After the fragmentation of each amino acid, to calculate the interaction energy between the receptor and the ligand, two methods were applied: density functional theory (DFT) formalism [34] and the generalized gradient approximation (GGA) functional B97D, computed by Gaussian 16 software package. It has been previously suggested that this GGA-type density functional is an accurate and efficient QM method [35], especially when dispersion forces are an important element, often valid for large systems. To expand the Kohn–Sham orbitals for all electrons, the 6-311 + G(d,p) basis set was chosen, which is a triple split valence (valence triple-zeta) small basis set with an additional diffuse function (+) and polarization functions (d,p). The conductor-like polarizable continuum model (CPCM) was utilized to account for solvent effects in QM calculations, with the dielectric constant (ε) set to 10 and 40. All terms of Equation (1) were subjected to DFT calculations.

Studies have shown that the contribution of an amino acid to the binding energy decreases as it moves further away from the ligand. To avoid unnecessary calculations of interactions, we performed a convergence analysis on the total binding energy. Our aim was to limit the number of considered amino acids to only those closest to the binding site while excluding the most distant ones. We achieved convergence criteria when the total energy, by increasing the radius r, did not change by more than 10% compared to the previous radius.

##### 2.5.6. In Silico ADMET Properties Analysis

An important computational step in the search for new drugs is the evaluation of the pharmacokinetic and druglikeness aspects of potential drugs. The SWISS-ADME [36] server is a powerful tool in the analysis of absorption, distribution, metabolism, excretion, and toxicity (ADMET). The server provides information on the ability to cross the blood–brain barrier (BBB), the gastrointestinal tract (GI), and possible violations of druglikeness rules such as the Lipinski rule. These and some other parameters were explored for the three molecules.

## 3. Results

### 3.1. Cytotoxicity Effects of Hops Fractions in Vero Cells

To evaluate the cytotoxic effect of hops fractions, we set the maximum concentration for toxicity assessment to 250 µg/mL of each one of four fractions tested, ranging from 250 µg/mL to 31.25 µg/mL. A 72 h post-treatment by a MTT cytotoxicity test assay as applied to analyze the Vero cell toxicity [37]. As previously described, the fractions displayed cell viability well above 50% at the concentration of 250 µg/mL and and lower concentrations tested [12]. The CC_50_ for all four fractions in this assay displayed cell viability above 50% at the highest concentration; thus, no CC_50_ value could be measured (Table 1 and Figure S1 in Supplementary Materials).

### 3.2. Hops Fractions Inhibition of OROV Replication Is Concentration-Dependent

**Figure 2:**
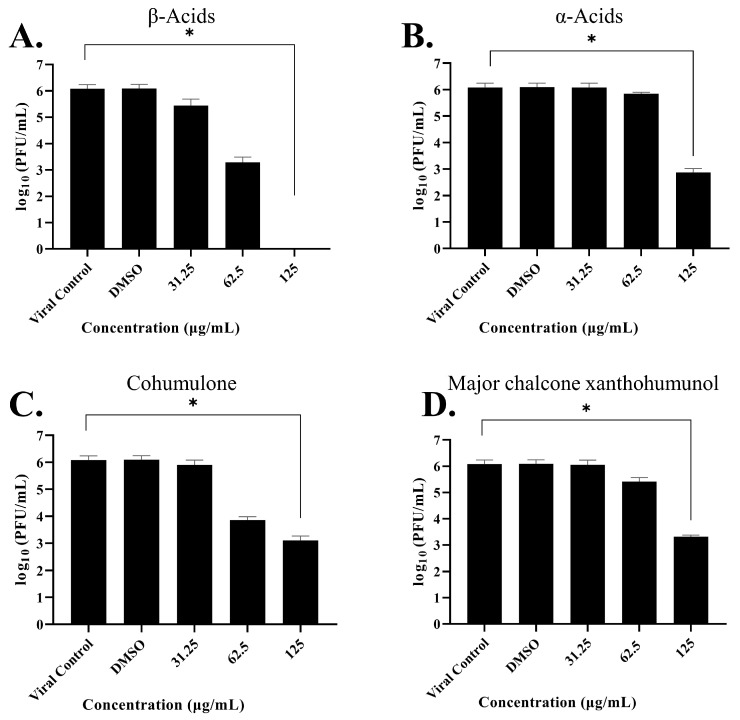
Hops fractions affect the viral yield in a concentration-dependent manner. OROV production was measured in the presence of variety of dilutions of the tested fractions in Vero cells, with preliminary inoculum of MOI 0.1 under post treatment. (**A**) β-Acids. (**B**) α-Acids. (**C**) Major α-Acid cohumulone. (**D**) Major chalcone xanthohumunol. The left vertical axis represents PFU infectivity titration of OROV. Standard deviations are represented by error bars. Three independent experiments gave the values for the mean ± standard error. Asterisks indicate statistical significance between each group and the control, as determined by Kruskal–Wallis test and subsequent Dunnett’s test (* *p* < 0.05).

To evaluate whether OROV progeny reduction is affected by hops fractions concentration, we treated Vero cells with different concentrations of the fractions, and the viral progeny production was measured. Interestingly, in the treatment of virus-infected cells during the entire post-inoculation period, we found a significant dose-dependent decrease in viral titers (Figure 2A–D) in all four fractions, with reductions in viral progeny yields in treated cells.

At fraction β-Acids (Figure 2A), we observed a viral progeny yields reduction at 125 µg/mL of up to 6.0 Log_10_ units compared to the viral control titers, whereas, at the 62.5 and 31.25 µg/mL concentrations, 2.79 and 0.67 Log_10_ units decreases were observed. An EC_50_ of 26.7 µg/mL was found, along with the selectivity index (SI) > 9.4 (calculated by CC_50_ value divided by the EC_50_ value).

At fraction α-Acids (Figure 2B), we observed a viral progeny yields reduction at 125 µg/mL of up to 3.21 Log_10_ units compared to the viral control titers, whereas, at the 62.5 µg/mL concentration, a 0.23 Log_10_ units decrease was observed. An EC_50_ of 65.6 µg/mL was found, and the selectivity index (SI) was 3.8.

At fractions with the major α-Acid cohumulone (Figure 2C), we observed a viral progeny yields reduction at 125 µg/mL of up to 2.94 Log_10_ units compared to the viral control titers, whereas, at the 62.5 µg/mL concentration, a 2.19 Log_10_ units decrease was observed. An EC_50_ of 34.3 µg/mL was found, and the selectivity index (SI) was 7.3.

At fractions with major chalcone xanthohumol and flavonoids (Figure 2D), we observed a viral progeny yields reduction at 125 µg/mL of up to 2.73 Log_10_ units compared to the viral control titers, whereas, at the 62.5 µg/mL concentration, a 0.63 Log_10_ units decrease was observed. An EC_50_ of 50.2 µg/mL was found, and the selectivity index (SI) was 4.9.

### 3.3. Hops Fractions Reduces OROV Progeny Yield

**Figure 3:**
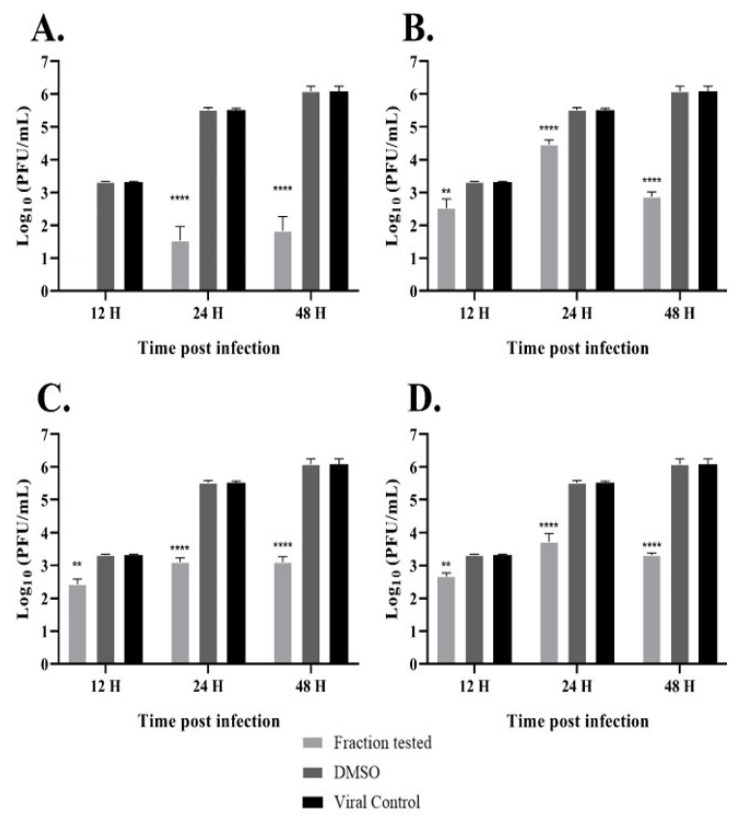
Hops fractions affect viral progeny production. Antiviral effects of hops fractions (125 µg/mL) against OROV (MOI = 0.1) infection. Vero cells were infected with OROV and treated with: (**A**) β-Acids; (**B**) α-Acids; (**C**) Major α-Acid cohumulone; (**D**) Major chalcone xanthohumunol. Culture supernatants were harvested at 12, 24, and 48 hpi, and OROV progeny yields were measured through plaque-forming assays. Values are the mean ± standard error obtained from three independent experiments. Asterisks indicate statistical significance between the control and each group, as determined by two-way ANOVA and subsequent Dunnett’s test (** *p* < 0.001 and **** *p* < 0.0001).

Furthermore, to evaluate the effects of hops fractions inhibitions of OROV replications, we measured viral progeny yields by plaque formation assays on the course of 12, 24, and 48 h post-infection. The results demonstrated that fractions at a concentration of 125 µg/mL promoted a more significant inhibition in viral titers from the first 12 h up to 48 h post-infection (Figure 3A–D), suggesting that fractions are able to impair OROV replication at an early stage of the viral cycle, and some significant inhibitory effects could still be observed even at the 48 h post-infection point.

### 3.4. Molecular Dynamics Simulation and Ensemble Docking

Prior to conducting molecular docking, we conducted 200 ns of MD simulations on the single experimental model of the N-terminal OROV endonuclease domain. The protein structure’s flexibility during the MD simulations was assessed by calculating the RMSD and RMSF (Figure S3). The active region responsible for coordinating the Mn^2+^ ion was previously identified as the binding region of the protein [1].

**Figure 4:**
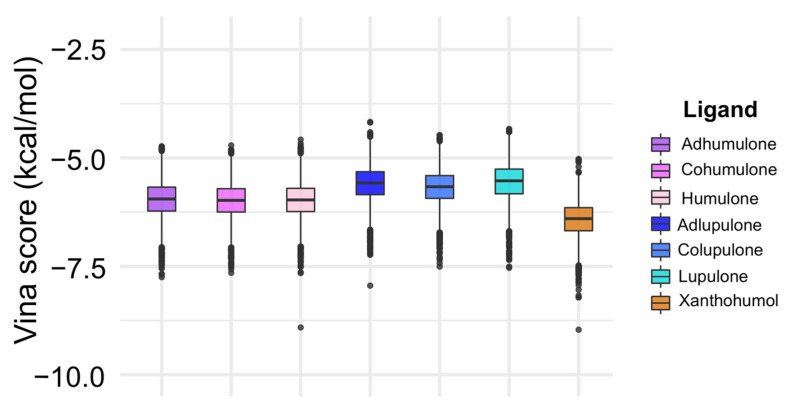
Boxplot of docking results for ligands in 2000 frames of OROV Endo-Nter domain. A complete version of this boxplot showing the outliers can be seen in Figure S2.

A boxplot depicting the energy distribution of each complex after docking is presented in Figure 4. The energy values of the complexes ranged from −31.135 kcal/mol to −4.170 kcal/mol, with the lowest and highest energy values being −31.135 kcal/mol to −7.75 kcal/mol and −5.023 kcal/mol to −4.170 kcal/mol, respectively. It is noteworthy that the energy values of alpha acids and beta acids were quite similar among them, with the former exhibiting a slightly higher affinity score. Xanthohumol, on the other hand, displayed a lower mean affinity score as compared to alpha and beta acids fractions. On average, the binding score could be ranked in the decreasing order of affinity: xanthohumol > alpha acids > beta acids. For further molecular dynamics (MD) simulation, one main representative of each group from alpha and from beta acids were selected. Therefore, the best complexes (with lower vina scores) of cohumulone (alpha acid), colupulone (beta acid), and xanthohumol were chosen. A summary of the docking results for all the complexes can be found in Table 2.

### 3.5. Molecular Dynamics Simulation and MM/PBSA Analysis

**Figure 5:**
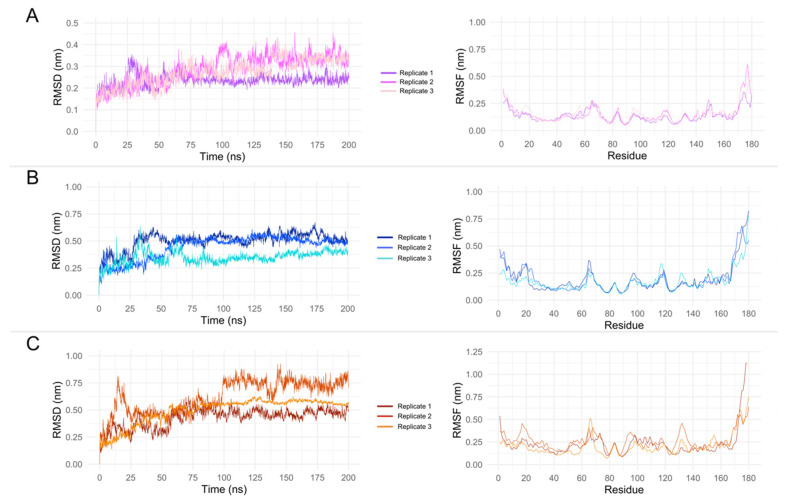
RMSD and RMSF of OROV Endo-NTer domain complexed with: (**A**) cohumulone, (**B**) colupulone, and (**C**) xanthohumol. All simulations were performed in three independent replicates. The RMSD and RMSF calculations were performed considering the protein backbone. RMSD of ligand can be seen in Figure S3.

The root mean square deviation (RMSD) analysis of the protein–ligand complexes showed different stability patterns among replicates. The cohumulone complex exhibited the most stable behavior in replicate 1, with RMSD values ranging from 0.2 nm to 0.3 nm after 100 ns of simulation. In replicates 2 and 3, higher fluctuations were observed, with values ranging from 0.25 nm to 0.45 nm after 125 ns (Figure 5A). Despite the difference in RMSD behavior, the RMSF (Figure 5A) indicated no significant difference among the replicates except for the C-terminal portion (residues 170 to 180), where replicates 2 and 3 displayed higher fluctuations compared to replicate 1.

For the colupulone complexes, the RMSD of the trajectory revealed distinct patterns of structural stability among the replicates. Replicate 1 exhibited high fluctuations, with RMSD values ranging from ~0.45 nm to 0.67 nm even after 125 ns of simulation. Conversely, replicates 2 and 3 displayed greater stability, with RMSD values ranging from 0.32 nm to 0.46 nm and 0.44 nm to 0.57 nm, respectively, after 150 ns of simulation (Figure 5B). Analysis of the RMSF plot (Figure 5B) revealed that the N- and C-terminals exhibited the most pronounced differences in fluctuation among the replicates.

The RMSD analysis of xanthohumol complexes also showed distinct patterns among replicates. The lowest RMSD values were observed in replicate 1, although some fluctuations were observed, with values ranging from 0.38 nm to 0.50 nm. In contrast, replicate 2 exhibited higher RMSD values, particularly after 100 ns of simulation, with values ranging from 0.56 nm to 0.92 nm. Replicate 3 displayed an intermediate RMSD value, with a narrow range of values (0.53 nm to 0.62 nm) and a standard deviation of 0.02 (Figure 5C). The RMSF plot revealed that replicate 1 had higher fluctuations in the C-terminal portion, whereas replicate 2 exhibited higher fluctuations near residues 17–19 and 130–132 (Figure 5C). A summary of the results of the MD simulations is presented in Supplementary Table S1.

The observed differences among replicates are an important result to evaluate a different conformation of a protein complexed with a ligand. However, it is important to highlight that the ligands were maintained in the binding pocket during all simulations (See Figure S3). As the observed fluctuations were mainly in the N- and C-terminals, the MM/PBSA analysis were performed for all replicates and focused on the last 50 ns (500 frames). 

The results of the MM/PBSA analysis are presented in Table 3. In all replicates and ligands, a negative average value of binding energy was observed, except for replicate 2 of colupulone, where the mean was 1.94 kcal/mol. The lowest value of binding energy ranged from −9.43 kcal/mol to −25.37 kcal/mol. The complexes with the highest affinity for cohumulone, colupulone, and xanthohumol were observed in replicate 2 (−16.70 kcal/mol), replicate 1 (−16.07 kcal/mol), and replicate 3 (−25.37 kcal/mol), respectively. These complexes were subsequently subjected to QM analysis. The plot of binding energy by frame of MM/PBSA analysis can be seen in Figures S4–S6.

### 3.6. Quantum Interaction Analysis of OROV Endo-NTer Complexed with Cohumulone, Colupulone, and Xanthohumol

**Figure 6:**
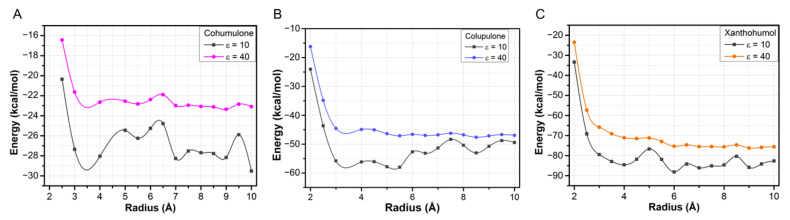
Total interaction energy (in kcal/mol) of the complexes (**A**) cohumulone, (**B**) colupulone, and (**C**) xanthohumol as a function of binding pocket radius distance (in Å). Two dielectric constants were settled (ε = 10 and ε = 40) for each complex.

Each complex that exhibited the lowest binding affinity for cohumulone, colupulone, and xanthohumol was subjected to QM energy calculation. QM calculation is a highly accurate approach for analyzing intermolecular interactions in protein–ligand complexes and has been widely used for this purpose [38,39,40]. Figure 6 displays the cumulative binding energy as the radius increases. For the cohumulone complex (Figure 6A), the convergence criteria are not met when ε = 10, and the radius is 10 Å. However, it is worth noting that, in all complexes, the cumulative energy for ε = 10 is lower than for ε = 40. This suggests that the calculations were accurately performed since weaker inter-molecular interactions are expected as ε increases.

The convergence criteria were met for the colupulone, cohumulone, and xanthohumol complexes at 6 Å, 7.5 Å, and 6.5 Å for ε = 40, respectively. The calculation was performed up to a radius of 10 Å to ensure all the important residues were analyzed. The calculated energy values for ε = 40 (ε = 10) were as follows: cohumulone −23.74 kcal/mol (−29.52 kcal/mol), colupulone −46.96 kcal/mol (−49.43 kcal/mol), and xanthohumol −75.60 kcal/mol (−82.67 kcal/mol), indicating that xanthohumol had the highest affinity for OROV Endo-Nter protein followed by colupulone and cohumulone. This result contrasts with that observed for docking and MM/PBSA analysis, where cohumulone was found to have a higher affinity than colupulone.

**Figure 7:**
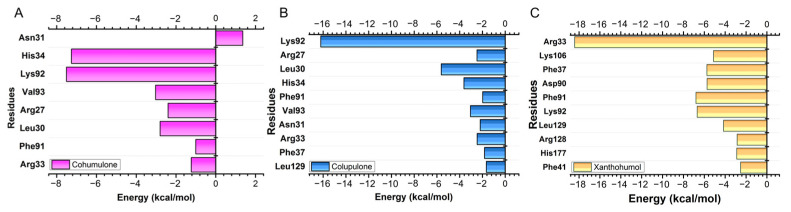
Horizontal bar plot showing the residues that most contributed to the interaction energies of the complexes (**A**) cohumulone, (**B**) colupulone, and (**C**) xanthohumol.

The MFCC approach offers the advantage of reducing the computational cost of QM calculations while allowing for per-residue analysis of binding energy. Figure 7 illustrates the main residues that contributed to the binding energy, and the graphic panels show the results achieved for the values in the dielectric constant ε = 40. In the case of cohumulone, the residues with higher affinity were Lys92 (−7.51 kcal/mol), His34 (−7.26 kcal/mol), Val93 (−3.03 kcal/mol), Leu30 (−2.80 kcal/mol), Arg27 (−2.40 kcal/mol), Arg33 (−1.23 kcal/mol), and Phe91 (−1.01 kcal/mol). However, the Asn31 residue presented a repulsive energy (1.90 kcal/mol). For colupulone, the residues that were highlighted as most important for protein binding were Lys92 (−16.21 kcal/mol), Leu30 (−5.62 kcal/mol), His34 (−3.62 kcal/mol), Val93 (−3.05 kcal/mol), Arg27 (−2.48 kcal/mol), Arg33 (−2.46 kcal/mol), and Asn31 (−2.19 kcal/mol). Repulsive interactions between proteins and ligands were not observed. Unlike cohumulone and colupulone, the most important residue for xanthohumol was Arg33 (−18.42 kcal/mol) following Lys92 (−6.68 kcal/mol), Lys106 (−5.12 kcal/mol), Phe91 (−6.80 kcal/mol), Phe37 (−5.75 kcal/mol), Arg128 (−2.85 kcal/mol), and Leu129 (−4.16 kcal/mol). In all complexes, Lys92, Phe91, and Arg33 have been presented as important residues for protein–ligand interactions. 

**Figure 8:**
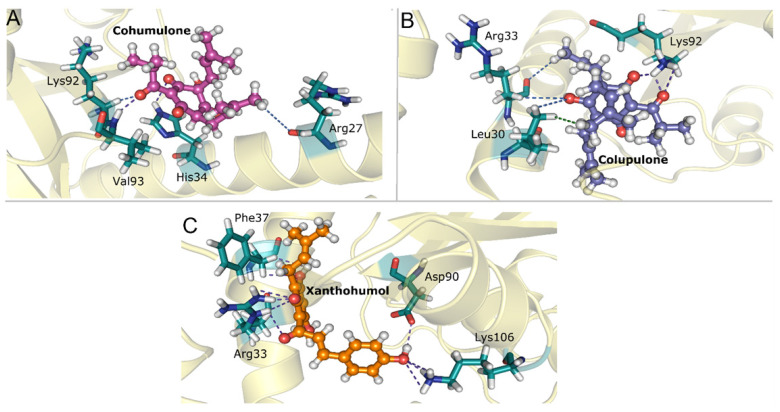
Illustrative representation of interactions with main residues for the protein-ligand complex. (**A**) Intermolecular interactions between Cohumulone and residues Val93, His34, Lys92 e Arg27. (**B**) Intermolecular interactions between Colupulone and residues Arg33, Lys92 and Leu30. (**C**) Intermolecular interactions between Xanthohumol and residues Asp90, Lys106, Arg33 and Phe37. The dashed lines refer to H-bonds (purple), non-conventional H bonds (blue) and alkyl-alkyl (green).

Figure 8 represents some of the main interactions involved in each protein–ligand system. The interactions demonstrated for cohumulone (Figure 8A) denote that residues Val93 and His34 present hydrogen bonds with the ligand, whereas Lys92 and Arg27 exhibit non-conventional hydrogen bonds. For the colupulone ligand (Figure 8B), the Arg33 residue shows a non-conventional H-bond, whereas Lys92 exhibits H-bond interactions, and Leu30 interacts with the ligand through alkyl–alkyl interactions. The illustrative representations observed in Figure 8C, which show the interactions between xanthohumol, demonstrate that Asp90, Lys106, Arg33, and Phe37 present a network of H-bond interactions with the ligand.

### 3.7. Alanine Scanning

To evaluate the impact of two shared residues on the binding energy of all three complexes, we conducted an alanine scanning analysis using the gmx_MMPBSA 1.6.1. program for residues Lys92 and Arg33, both important for all complexes. We selected the replicates with the lowest binding energy for each complex, specifically replicate 2 for cohumulone, replicate 1 for colupulone, and replicate 3 for xanthohumol. The results of this analysis can be found in Table 4. It has been noted that, for K92A mutation, all binding affinity has been reduced. For R33A, the affinity increased in the cohumulone complex.

### 3.8. ADMET Properties Analysis

It is essential to consistently factor in ADMET properties during the development of a novel medication, as they play a pivotal role in establishing the appropriate dosage, assessing toxicity, determining the administration route, and setting the frequency of dosing to better predict some pharmacokinetic properties of small molecules; it is interesting to evaluate some of these properties based on their structures in silico. For this purpose, the online server SwissADME was used to predict various characteristics of the three molecules. Six main characteristics are evaluated in the radar plot (See Supplementary Figure S7): flexibility, lipophilicity, size, unsaturation, polarity, and insolubility. Among them, only cohumulone showed a lipophilicity slightly beyond the suitable for drugs, whereas xanthohumol was out of the parameter of number of unsaturation (Figure S6). Despite this, this discrepancy was minimal for both molecules. When the ability to penetrate the blood–brain barrier (BBB) was evaluated, all molecules were unable to penetrate, indicating that a neurological effect is unlikely for these compounds.

The three molecules showed high gastrointestinal (GI) absorption, and only xanthohumol did not show as a substrate of P-gp, which indicates that it may have higher bioavailability compared to cohumulone and colupulone, which show as substrates of P-gp (Table 5). Regarding the inhibition of CYP isoforms (CYP1A2, CYP2C19, CYP2C9, CYP2D6, and CYP3A4), it is possible to observe that cohumulone showed important interactions with most of them. It is known that the inhibition of these CYP isoforms can lead to undesirable effects of drug accumulation since more than 90% of drugs are substrates of CYP isoforms, which, in turn, are important for drug clearance by metabolic biotransformation. Each of the three examined substances displayed inhibitory effects on CYP3A4. CYP3A4 serves as the primary enzyme responsible for the initial metabolic processing (phase I metabolism) of various substances such as steroid hormones, lipids, and bile acids. Additionally, it plays a crucial role in metabolizing foreign substances, including components from our diet and more than half of all prescription medications. 

In the evaluation of druglikeness, cohumulone did not show any violation in the rules evaluated by the server (Lipinski, Ghose, Veber, Egan, and Muegge), xanthohumol only violated the rule of Muegge [41] (pharmacophore point filter), and colupulone only met the rules of Lipinski and Veber [42] (oral bioavailability measurements). Thus, based on the pharmacokinetic and druglikeness properties, the three molecules showed positive and negative points, requiring further evaluations to define the most promising molecule as a drug candidate.

## 4. Discussion

**Figure 9:**
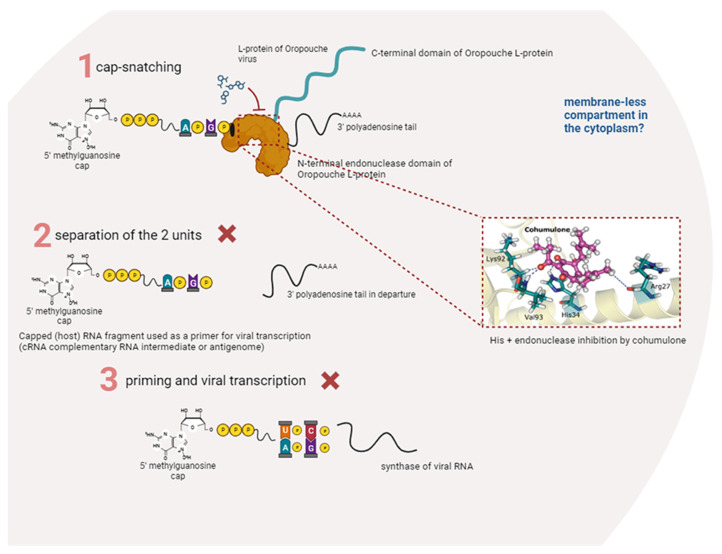
Illustrative representation of schematic overview about bunyavirus transcription. In the red rectangle, the probable location of the hops compound inhibition is represented, and cohumulone is used as example. The “red x” indicates all inhibited steps due to ligand–protein interactions.

Bunyaviruses, negative-sense, or ambisense single-strand RNA viruses can infect a diverse array of hosts, including vertebrates, invertebrates, and plants. WHO lists three bunyavirus diseases as priority diseases requiring urgent development of medical countermeasures, highlighting their high epidemic potentials [43]. The urgent need for effective antiviral treatments for emerging viruses necessitates a comprehensive approach that considers holistic understanding of the challenge. It is important to classify and analyze enzymes across viral phylogeny to avoid addressing individual viral spillovers while potentially overlooking crucial pathogens with outbreak potential. At the same time, natural extracts offer a range of small compounds that can be utilized in clinical drug development, particularly in the context of treating infectious diseases [44]. Li et al. [45] identified potent inhibitors against another Bunyavirales species after high-throughput screening (HTS) of small molecular compound library with 1058 compounds, derived from natural products. The major compound toosendanin, a triterpenoid saponin extracted from the fruit of *Melia toosendan* Sieb et Zucc (*Meliaceae*), was identified to have anti-viral effect. Severe fever with thrombocytopenia syndrome virus (SFTSV), a tick-borne virus, was inhibited both in vitro and in vivo on viral internalization, in the early stage of infection. In addition, another Bunyavirus—Rift Valley fever virus (RVFV)—was inhibited in the entry step, as well. Furthermore, toosendanin was reported to suppress the hepatitis C virus signaling pathway through the enhancement of alpha interferon and inhibited influenza A virus replication by interfering the nuclear localization of the viral polymerase PA protein. It was suggested that the triterpenoid saponin is an agonist of L-type voltage-dependent calcium channels, which might participate in the regulation of intracellular calcium homeostasis. In another study, Geerling et al. [46] evaluated the antiviral potential of natural based chemotypes through screening. In addition, form the α-hydroxytropolones (αHTs), metal-chelating motifs, or motifs similar to metal-chelating ones, *β*-thujaplicinol was active, with an EC_50_ of 13.8 μM, against RFVF. The αHTs are known as well to work against the HIV integrase or ribonuclease H by the metal-chelating mechanism [47]. The current classification of antiviral targets and guidelines of mechanisms of antivirals may need to be reevaluated, moving away from a case-by-case approach towards a classification based on viral families. In this context, we studied the in silico potential of endonuclease, a conserved global architecture protein within the L-protein of Bunyavirales and with vast equivalence to the influenza polymerase endonuclease [2,33]. Bunyaviruses replicate in the cell cytoplasm; however, the exact locations of bunyavirus genome replication and transcription are unknown. All bunyavirus endonucleases harbor a PD(E/D)K active site motif and coordinate two divalent metal ions crucial for catalytic activity. In addition, these endonucleases have been classified as either His– or His+, depending on whether or not they have a histidine upstream of the active site sequence motif that coordinated the first cation. Oropouche virus should be classified as “His+” by being a *Peribunyaviridae* member, after the work of Olschewski et al. [48] This was confirmed by our dynamic modulation results, where His34 gives important ligand–protein interaction in the case of the two acylphloroglunols cohumulone and lupulone, different from xanthohumol, where this acid is not identified as crucial. Schematic representation of the cap-snatching mechanism for Bunyavirales and, in particular, the Oropouche virus by the inhibition of one of the tested compounds is presented in Figure 9. The endonuclease domain is essential for virus replication and presents a structurally conserved active site that can be exploited to generate broad-spectrum antivirals. It is important to note that most of the endonuclease inhibitors for antiviral drug discovery implicitly target the two metal-binding sites of the endonuclease, which are also the targets for many HIV integrase inhibitors [49]. In this context, metal-chelating molecules have been extensively explored as potential inhibitors of viral metal-dependent enzymes, resulting in some important classes of antiviral agents as diketo acids [7].

Acylphloroglucinols contain chemical features worth exploring in the design of novel inhibitors. Further analysis needs to confirm the common pharmacophore that could emerge: the key interaction of those acylphloroglucinols with the Mn^2+^ ions through the carbonyl group and if it follows a chelation geometry [9]. However, beta acids are distinguished from alpha acids by having an additional isoprenyl chain instead of a hydroxyl group in position C4. This means that alkyl groups tend to contribute electrons to the carbonyl group, resulting in a stronger inductive effect with a positive influence (+I effect) and reducing the electronegativity of the carbonyl group. On the other hand, the hydroxyl group of the alpha acids, which occupy the same position, exhibit an electron-withdrawing inductive effect from the hydroxyl group, thereby increasing the electronegativity of the neighboring carbonyl group. As a result, the carbonyl group from the acyl side chain, together with the hydroxyl group from the benzyl ring, could form a chelating core and chelate metal ions at the active center, therefore inhibiting enzymatic reactions. Based on the cell-based assay conducted on Vero cells, the compounds’ EC_50_ values (µg/mL) in decreasing activity order are as follows: beta acids (26.7) > cohumulone (34.3) > xanthohumol and flavonoids (50.2) > alpha acids (65.6). At the highest concentration tested (125 µg/mL), the fractions showed significant inhibition of viral titers from 12 to 48 h post-infection. This indicates that the fractions can hinder OROV replication at an early stage of the viral cycle. Even at the 48 h post-infection point, considerable inhibitory effects were still evident. Among all the compounds, beta acids demonstrated the strongest activity, suggesting their potential to inhibit various steps of the OROV replication cycle, including genome translation, replication, virion assembly, and virion release from the cells. On the other hand, during the molecular dynamic simulation of endonuclease participating in the transcription, RNA binding, and cleavage, it was observed that the compounds have the capability to hinder the viral replication cycle, and xanthohumol demonstrated a lower mean affinity score compared to the alpha and beta acids fractions. On average, the ranking of binding affinity was as follows: xanthohumol > alpha acids > beta acids. The root mean square deviation (RMSD) analysis of the protein–ligand complexes showed different stability patterns among the three replicates. The cohumulone complex exhibited the most stable behavior in replicate 1. The quantum mechanical (QM) approach showed that xanthohumol had the highest affinity for the OROV endonuclease protein followed by colupulone and cohumulone. This result contrasts with that observed for docking and MM/PBSA analysis, where cohumulone was found to have a higher affinity than colupulone. The main residues identified as important for the ligand–protein interaction were Lys92 and Arg 33 for all three ligands. In the case of cohumulone, the residues with higher affinity were Lys92, His34, Val93, Leu30, Arg27, Arg33, and Phe91; for colupulone, the residues that were highlighted as most important for protein binding were the same, with Phe91 absent. Furthermore, the residue His34, identified for both alpha and beta acids and not for xanthohumol, was established as important residues for the active site and the binding of one of the two manganese ions of La Crosse virus (LACV) endonuclease (LC180), the same family as Oropouche virus [50]. Regarding xanthohumol, the residues that presented as important were Arg33 followed by Lys92, Lys106, Phe91, Phe37, Arg128, and Leu129. In all complexes, Lys92 presented as an important residue for protein-ligand interactions. Lys92 (together with Lys106) is known as one catalytic lysine important for endonuclease activity. The mutational analysis of the nuclease activity of OROV polymerase N-terminal domain confirmed the importance of both Lys92 and Arg33. We made a series of alanine point mutants of the two key conserved residues in order to assess their importance for activity. The K92A mutation led to a decrease in the binding affinity for all interactions. However, in the case of the R33A mutation, there was an increase in the affinity, specifically in the cohumulone complex. The substitution of a polar amino acid with a non-polar Ala residue resulted in a decrease in activity. RNA binding is typically facilitated by positively charged side chains and polar residues such as Arg, which has tendencies to form hydrogen bonds with RNA. This change could account for the observed reduction in activity. Nevertheless, our study has some limitations, as it was not possible to perform the binding assay of the compounds on our target protein in vitro, and the tests conducted were only in non-human mammalian cells. The limitation of the in silico study lies in the fact that the study used a protein model generated by an online server, which resulted in a model missing a Mn^2+^ ion since there is no experimentally resolved structure. The MD, in turn, was limited only to classical mechanics methods, not being able to observe possible relevant chemical processes, such as formation or breaking of bonds. Moreover, the study proposes Endo-Nter as a possible target for action against OROV, not taking into account other viral proteins.

## 5. Conclusions and Future Perspectives

From a clinical standpoint, the current approach to treating OROV infection primarily concentrates on alleviating symptoms rather than directly targeting the virus. The focus is on providing relief from symptoms without directly attacking or eradicating the causative agent, such as by inhibiting the virus’s ability to replicate or by directly killing it. Despite afflicting over half a million people in South America, the disease, much like other arboviral illnesses, has been neglected for years. In our current investigation, we identified three categories of potential antiviral treatments against the OROV virus from the broader class of pathogenic viruses known as the Bunyavirales order. These three groups of compounds belong to the acylphloroglucinols and prenylated chalcones/phenolic compound classes. We verified that, in the case of this particular species, a histidine upstream is present on the active site sequence motif that coordinates the first manganese cation during the transcription step by a cap-snatching mechanism. Computational modeling studies allowed us to define two key conserved residues, and we assessed their importance for binding activity with the tested compounds. Enhancing the experimental model of the N-terminal endonuclease structure with the inclusion of two Mn^2+^ ions, in the absence of a ligand, could serve as confirmation of the inhibitory potential through a chelation mechanism of hops compounds. However, the results can only be confirmed with structural studies. Our study is the first to provide a detailed description of the Oropouche viral strain in the presence of monocyclic polyprenylated acylphloroglucinols and a chalcone. We investigated the viral progeny yields in vitro of interesting candidates as lead structures for drug development by their highly oxygenated derived cores decorated with side prenylated chains. At the highest concentration tested of 125 µg/mL, all fractions showed significant inhibition of viral titers from 12 to 48 h post-infection. Complementary studies are needed to comprehend the mechanism in a deeper level; however, the current work unquestionably suggests that these compounds have the capability to reduce OROV’s ability to infect host cells.

## Figures and Tables

**Table 1:** Hops compounds cell toxicity on Vero cell lineages.

**Concentration**	***β*-Acids**	***α*-Acids**	**Cohumulone**	**Xanthohumol and Flavonoids**
Cell Viability (%) *				
**250 µg/** **mL**	97.6 ± 15.8	90.0 ± 11.9	90.9 ± 11.4	96.4 ± 12.1
**EC_50_ (µg/** **mL)**				
	26.7	65.6	34.3	50.2
**SI (ratio of the CC_50_ to the EC_50_)**				
	>9.4	>3.8	>7.3	>4.9

* Values with confidence intervals (CI) 95%.

**Table 2:** Summary of docking results.

Ligand/Fraction	Min.	1st Quartile	Median	Mean	3rd Quartile	Máx.	Sd ^†^
Adhumulone	−7.750	−6.224	−5.946	−5.995	−5.673	−4.731	0.4123
Cohumulone	−31.135	−6.247	−5.978	−5.994	−5.709	−4.711	0.5177
Humulone	−8.910	−6.236	−5.968	−5.978	−5.701	−4.578	0.3983
Adlupulone	−12.832	−5.846	−5.577	−5.595	−5.318	−4.170	0.4184
Colupulone	−7.506	−5.931	−5.665	−5.681	−5.407	−4.473	0.3977
Lupulone	−15.655	−5.827	−5.528	−5.557	−5.258	−4.332	0.4464
Xanthohumol	−8.962	−6.679	−6.400	−6.421	−6.147	−5.023	0.4105
Alpha acids ^⸸^	−31.135	−6.238	−5.963	−5.976	−5.695	−4.578	0.4462
Beta acids ^⸹^	−15.655	−5.813	−5.593	−5.611	−5.329	−4.170	0.4245

^†^ Standard deviation. ^⸸^ Adhumulone, cohumulone, and humulone. ^⸹^ Adlupulone, colupulone, and lupulone.

**Table 3:** MM/PBSA energy results in kcal/mol.

Ligand	Replicate 1		Replicate 2		Replicate 3	
	Mean ± Sd ^†^	Lowest Value	Mean ± Sd ^†^	Lowest Value	Mean ± Sd ^†^	Lowest Value
Cohumulone	−7.60 ± 2.75	−13.32	−8.98 ± 2.22	−**16.70**	−7.75 ± 2.75	−15.24
Colupulone	−7.21 ± 3.53	−**16.07**	1.94 ± 3.55	−9.43	−5.32 ± 2.80	−9.99
Xanthohumol	−5.95 ± 3.82	−18.19	−5.26 ± 2.49	−12.38	−16.22 ± 3.44	−**25.37**

^†^ Standard deviation.

**Table 4:** MM/PBSA energy results in kcal/mol after mutation.

Ligand	K92A		R33A		E48A	
	Mean ± Sd ^†^	ΔΔH ^&^	Mean ± sd ^†^	ΔΔH ^&^	Mean ± sd ^†^	ΔΔH ^&^
Cohumulone	−8.12 ± 0.61	0.86	−9.39 ± 2.17	−0.42	−9.0 ± 2.22	−0.02
Colupulone	−4.31 ± 3.40	2.90	−7.14 ± 3.46	0.07	−7.41 ± 3.53	−0.20
Xanthohumol	−13.47 ± 3.41	2.75	−9.30 ± 3.29	6.92	−16.46 ± 3.44	−0.24

^†^ Standard deviation. ^&^ Difference of binding energy (wild type—mutant).

**Table 5:** Pharmocokinetics and drug likeness properties for the three studied compounds, according to SwissADME server.

Properties	Parameters/Rules	Cohumulone	Colupulone	Xanthohumol
Pharmacokinetics	GI absorption	High	High	High
	BBB permeant	No	No	No
	P-gp substrate	Yes	Yes	No
	CYP1A2 inhibitor	No	No	Yes
	CYP2C19 inhibitor	Yes	Yes	No
	CYP2C9 inhibitor	Yes	No	Yes
	CYP2D6 inhibitor	Yes	No	No
	CYP3A4 inhibitor	Yes	Yes	Yes
Druglikeness	Lipinski	Yes	Yes	Yes
	Ghose	Yes	No	Yes
	Veber	Yes	Yes	Yes
	Egan	Yes	No	Yes
	Muegge	Yes	No	No

## Data Availability

The data presented in this study are available in this article.

## Supplementary Materials

The following supporting information can be downloaded at: https://www.mdpi.com/article/10.3390/pharmaceutics15122769/s1. Table S1. RMSD summary of the last 100 ns for each MD simulation. Figure S1. Cytotoxicity of hops fractions on Vero cells. After different concentrations of hops fractions were applied to Vero cells for 72 h, cytotoxicity was evaluated using the MTT assay. All data are presented with error bars, and three independent experiments were performed. Figure S2. Boxplot of docking energy distribution showing all energies, including the outliers. Figure S3. RMSD plot of ligand through 200 ns trajectory. Maximum displacement of ligand was 0.04 nm which suggests, in all complexes they were maintaining in the binding pocket during all simulation time. Figure S4. MM/PBSA binding energy per frame of OROV Endo-NTer complexed with cohumulone. The analysis was done using the last 500 frames (50 ns) of trajectory. Figure S5. MM/PBSA binding energy per frame of OROV Endo-NTer complexed with colupulone. The analysis was done using the last 500 frames (50 ns) of trajectory. Figure S6. MM/PBSA binding energy per frame of OROV Endo-NTer complexed with xanthohumol. The analysis was done using the last 500 frames (50 ns) of trajectory. Figure S7. Swiss-ADME output results showing the radar plot and the boiled-egg diagram. The yellow region represents the high probability of crossing the blood-brain barrier and the white region indicates the probability of passive absorption by the GI. The blue dots indicate effective efflux by P-gp (PGP+) and the negative represents the role of non-substrate of P-gp (PGP-).
