# Supplementary material for: Antiviral Activity and Molecular Dynamics Simulation of Hops Compounds against Oropouche Virus (Peribunyaviridae)
Source: Pharmaceutics. 2023 Dec 13;15(12):2769. doi: 10.3390/pharmaceutics15122769 (PMC10747393; doi:10.3390/pharmaceutics15122769)
Supplement: Supplementary file 1 [file pharmaceutics-15-02769-s001.zip › pharmaceutics-2623357-supplementary.pdf]

Supplementary Materials

Antiviral Activity and Molecular Dynamics Simulation of Hops Compounds against Oropouche virus (*Peribunyaviridae*)

Tsvetelina Mandova [1,2,*,†], Marielena Vogel Saivish [3,4,†], Gabriela de Lima Menezes [5,†], Katyanna Sales Bezerra [5], Umberto Laino Fulco [5], Roosevelt Alves da Silva [6], Fernando Batista Da Costa [1] and Maurício Lacerda Nogueira [3,7]

1 AsterBioChem Research Team, School of Pharmaceutical Sciences of Ribeirão Preto, University of São Paulo, Ribeirão Preto 14040-020, SP, Brazil
2 Gilson Purification, 22 rue Bourseul, 56890 Saint Avé, France
3 Laboratórios de Pesquisas em Virologia, Departamento de Doenças Dermatológicas, Infecciosas e Parasitárias, Faculdade de Medicina de São José do Rio Preto, São José do Rio Preto 15090-000, SP, Brazil; marielenasaivish@gmail.com (M.V.S.)
4 Brazilian Biosciences National Laboratory, Centro Nacional de Pesquisa em Energia e Materiais (CNPEM), Campinas 13083-100, SP, Brazil
5 Bioinformatics Multidisciplinary Environment, Programa de Pós Graduação em Bioinformática, Universidade Federal do Rio Grande do Norte, Natal 59078-400, RN, Brazil; gabrieladelima1804@gmail.com (G.d.L.M.); umberto.laino@ufrn.br (U.L.F.)
6 Núcleo Colaborativo de Biosistemas, Universidade Federal de Jataí, Jataí 75801-615, GO, Brazil; roosevelt@ufj.edu.br
7 Department of Pathology, University of Texas Medical Branch, Galveston, TX 77555, USA
* Correspondence: tmandova@gilson.com
† These authors equally contributed to this work.

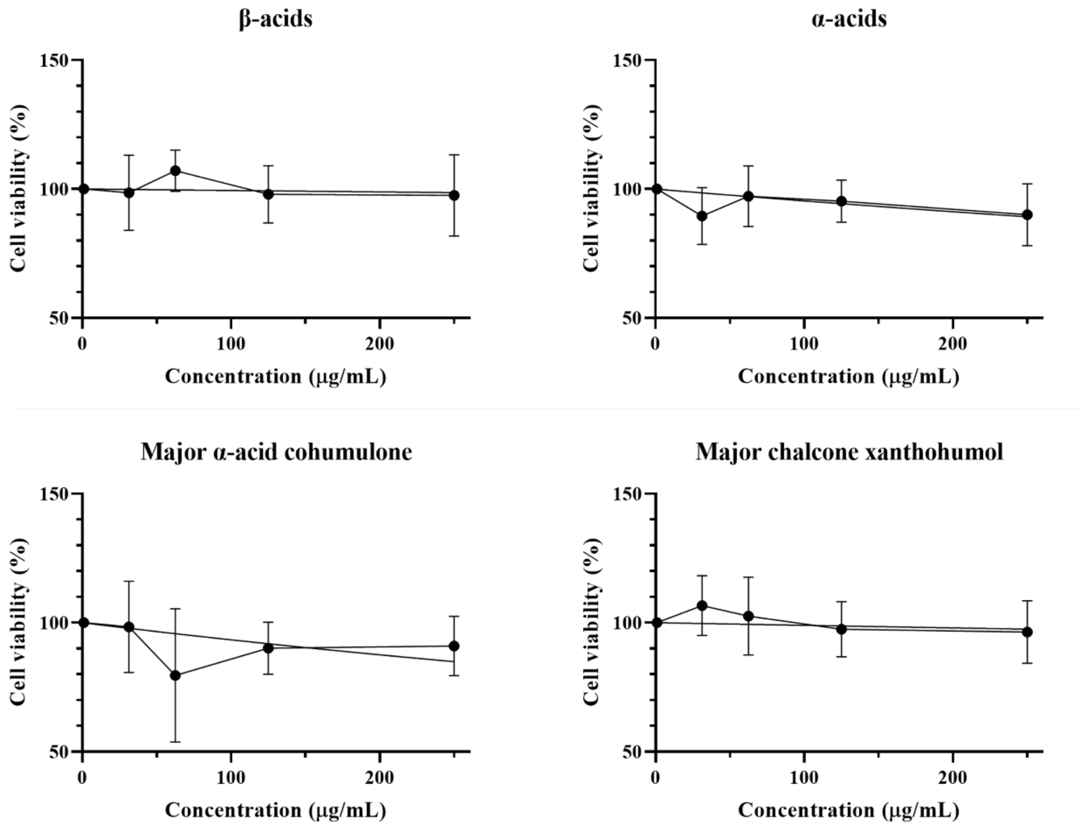

Figure S1. Cytotoxicity of hops fractions on Vero cells. After different concentrations of hops fractions were applied to Vero cells for 72 h, cytotoxicity was evaluated using the MTT assay. All data are presented with error bars, and three independent experiments were performed.

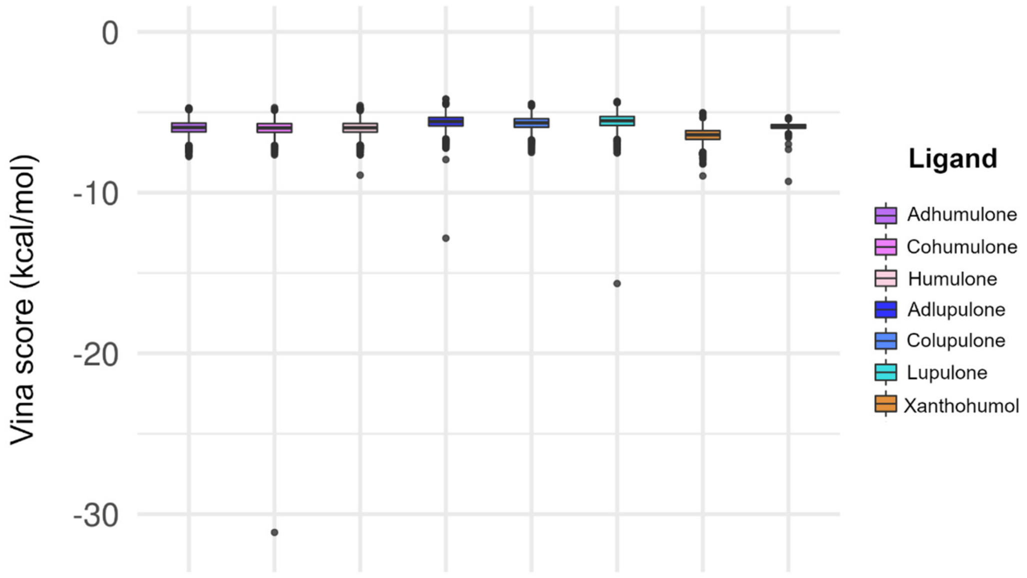

Figure S2. Boxplot of docking energy distribution showing all energies, including the outliers.

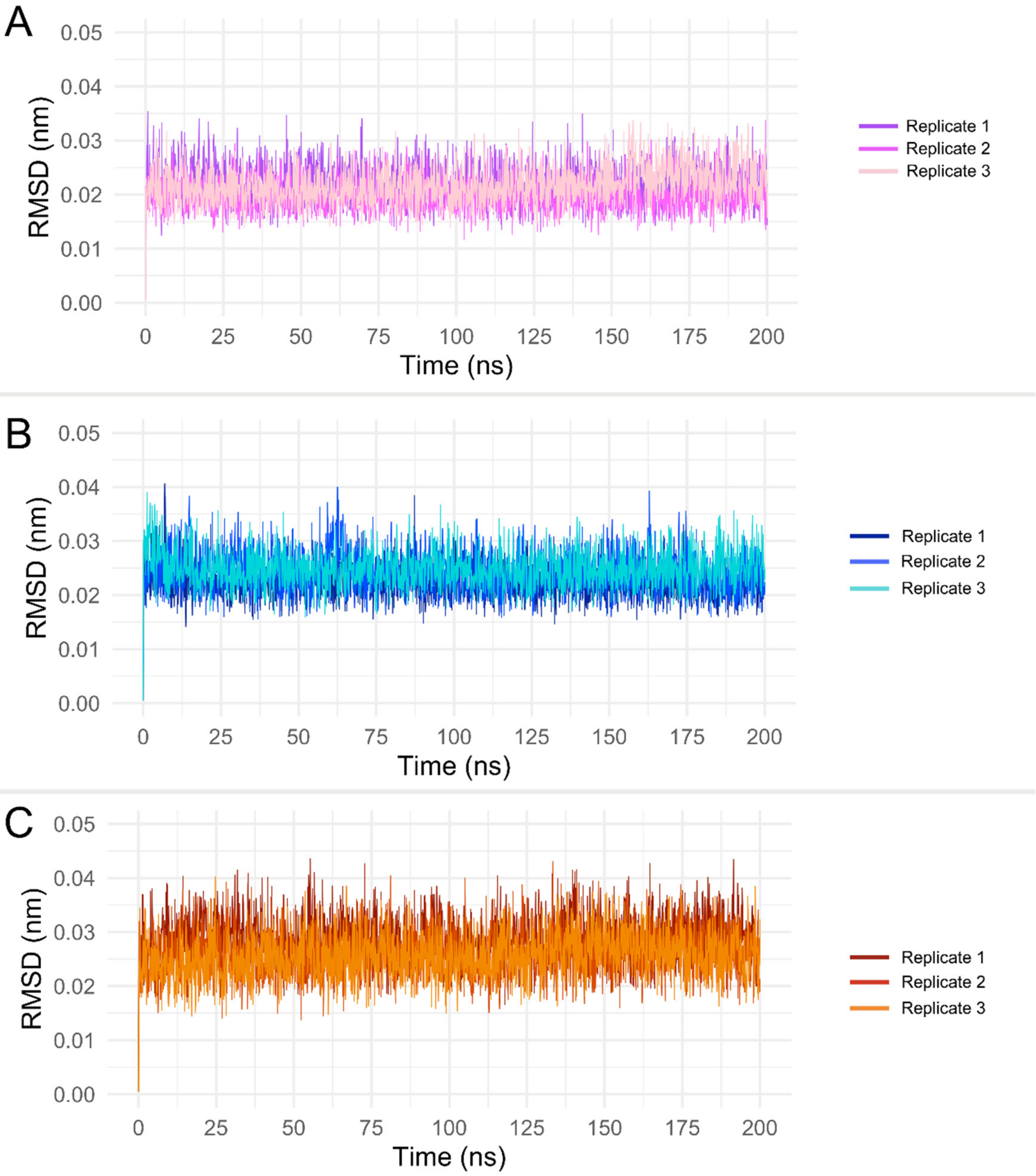

Figure S3. (**A–C**) RMSD plot of ligand through 200 ns trajectory. Maximum displacement of ligand was 0.04 nm which suggests, in all complexes they were maintaining in the binding pocket during all simulation time.

Table S1. RMSD summary of the last 100 ns for each MD simulation

Ligand (Replicate)	Min.	1st quartile	Median	Mean	3rd quartile	Máx.	Sd†
Cohumulone (1)	0.191	0.226	0.236	0.238	0.248	0.313	0.0426
Cohumulone (2)	0.223	0.314	0.333	0.336	0.355	0.456	0.0328
Cohumulone (3)	0.228	0.289	0.319	0.316	0.342	0.419	0.0342
Colupulone (1)	0.434	0.508	0.536	0.537	0.564	0.672	0.0418
Colupulone (2)	0.436	0.489	0.509	0.513	0.535	0.608	0.0296
Colupulone (3)	0.259	0.341	0.367	0.367	0.393	0.465	0.0351
Xanthohumol (1)	0.376	0.441	0.466	0.469	0.495	0.604	0.0367
Xanthohumol (2)	0.561	0.714	0.750	0.747	0.783	0.925	0.0542
Xanthohumol (3)	0.532	0.559	0.570	0.572	0.584	0.627	0.0182

Sd† = standard deviation

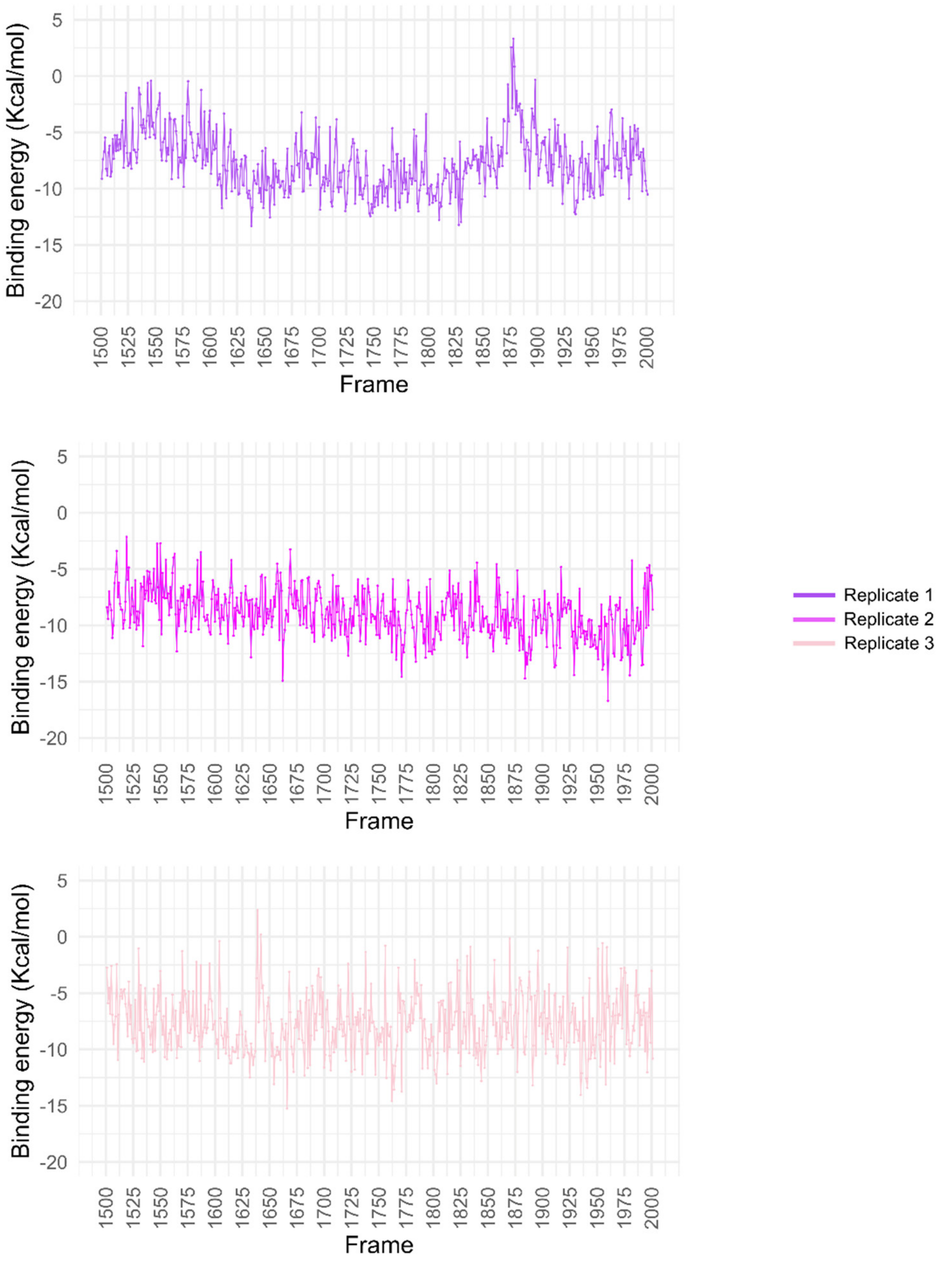

Figure S4. MM/PBSA binding energy per frame of OROV Endo-NTer complexed with cohumulone. The analysis was done using the last 500 frames (50 ns) of trajectory.

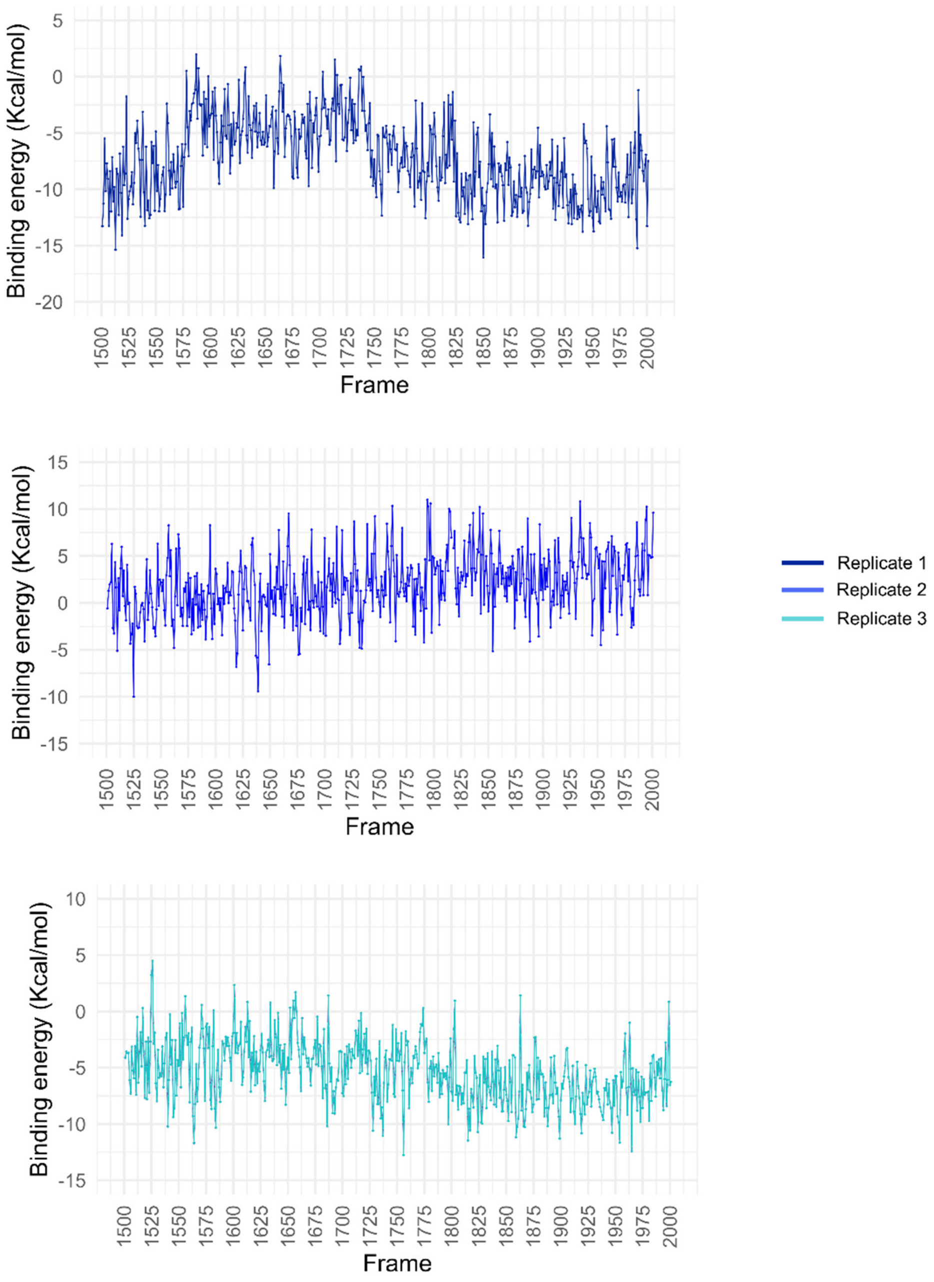

Figure S5. MM/PBSA binding energy per frame of OROV Endo-NTer complexed with colupulone. The analysis was done using the last 500 frames (50 ns) of trajectory.

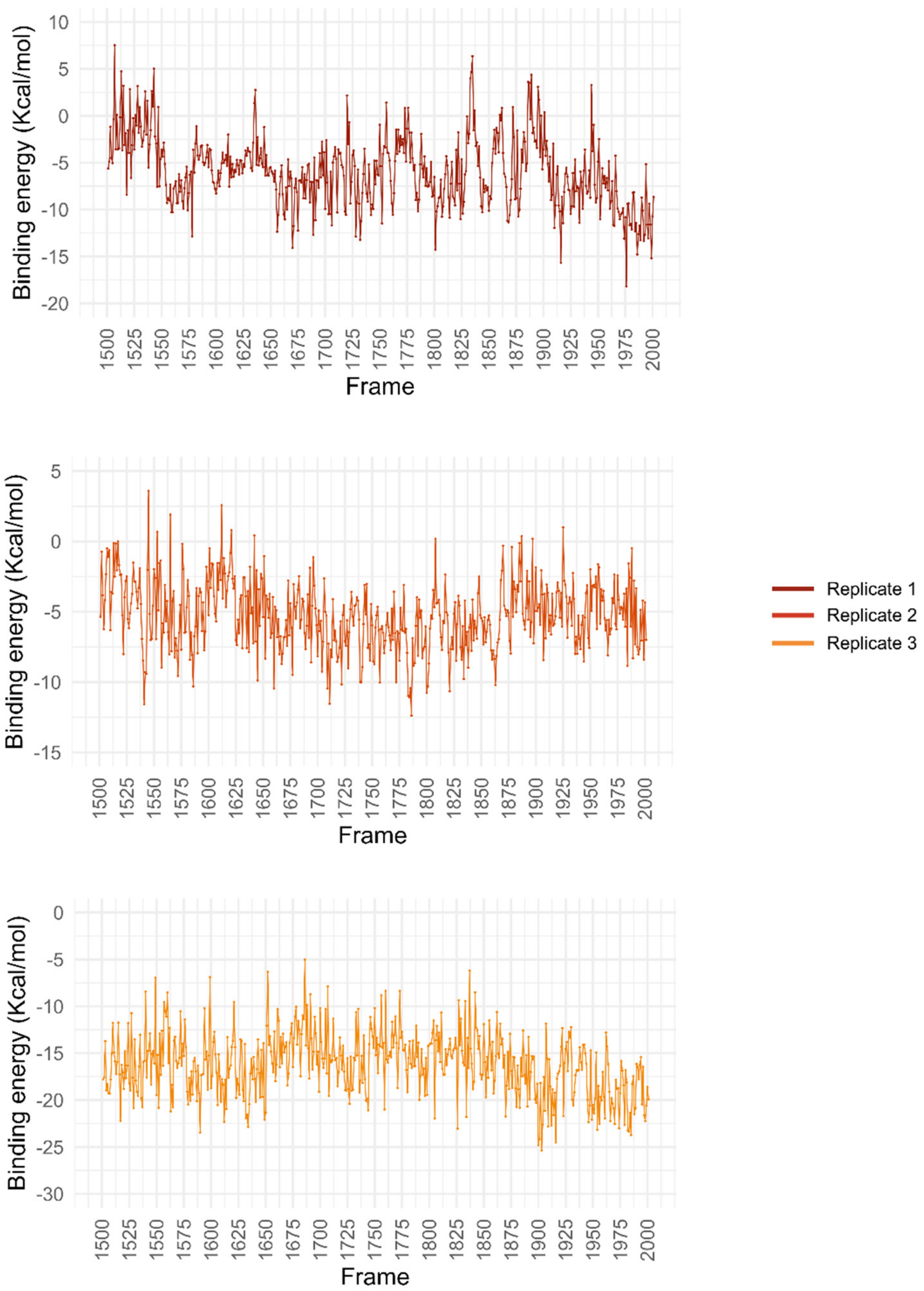

Figure S6. MM/PBSA binding energy per frame of OROV Endo-NTer complexed with xanthohumol. The analysis was done using the last 500 frames (50 ns) of trajectory.

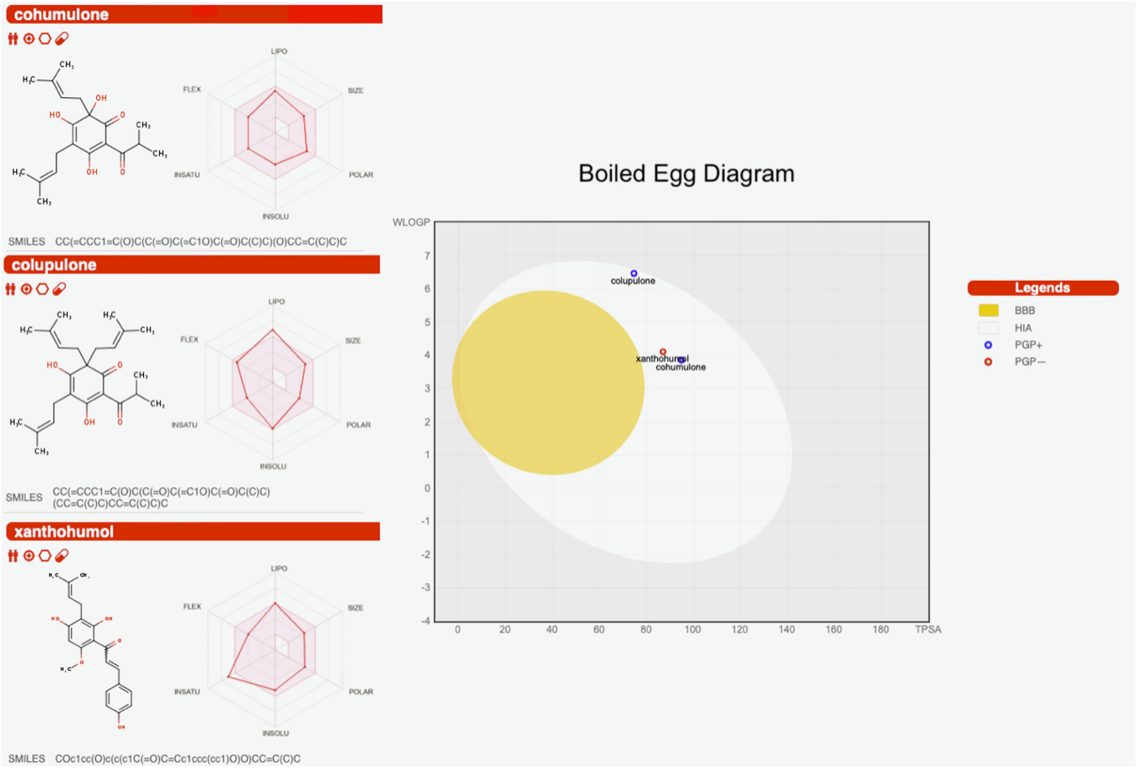

Figure S7. Swiss-ADME output results showing the radar plot and the boiled-egg diagram. The yellow region represents the high probability of crossing the blood-brain barrier and the white region indicates the probability of passive absorption by the GI. The blue dots indicate effective efflux by P-gp (PGP+) and the negative represents the role of non-substrate of P-gp (PGP-)
